# Existing Health Inequities in the Treatment of Advanced and Metastatic Cancers

**DOI:** 10.1007/s11912-024-01617-3

**Published:** 2024-11-04

**Authors:** Navya Nair, Matthew Schlumbrecht

**Affiliations:** 1 Division of Gynecologic Oncology, Sylvester Comprehensive Cancer Center, 1121 NW 14th St, Suite 345C, Miami, FL 33136 USA; 2 Department of Obstetrics, Gynecology, and Reproductive Sciences, University of Miami Miller School of Medicine, Miami, FL USA

**Keywords:** Cancer, Metastatic, Health inequity, Disparities, Race

## Abstract

**Purpose of Review:**

This study aims to identify health inequities related to the medical treatment and supportive care of patients with advanced/metastatic cancer and recommend solutions to promote health equity.

**Recent findings:**

Despite robust strides in the development of therapeutic strategies for advanced and metastatic cancer, significant disparities in treatment access and implementation exist. Race, socioeconomic status, gender, and geography represent just a few of the individual-level factors which contribute to challenges in treatment administration, thorough evaluation of germline genetics and tumor genomics, and quality palliative and end-of-life care.

**Summary:**

Given the increasing complexity of cancer treatments and our enhanced understanding of tumor biology, efforts to uniformly provide equitable and high-level care to all patients are needed. In this review we will discuss factors that contribute to health inequities in patients with advanced and metastatic cancer diagnoses, highlighting opportunities for intervention, ongoing challenges in change implementation, and national and international society recommendations to eliminate disparities. Acknowledging existing inequities and engaging in multilevel discourse with key stakeholders is needed to optimize care practices to the benefit of all patients.

## Introduction

Despite robust strides in the development of novel therapeutics for advanced and metastatic cancer, significant disparities in treatment access and implementation exist. The recognition of these disparities has improved significantly over the recent decades, and in a number of contexts, including screening, incidence, prevalence, stage, mortality, survival, morbidity, survivorship, and financial burden of the disease [1]. Description of these health inequities, however, has not translated into substantial systematic changes to eliminate them. Race remains a major contributor to health disparities. Black people are more likely than White people to die from cancer despite the increasing availability of effective treatments and declining national trends in cancer mortality, with marked disparities apparent specifically in malignances of the prostate, stomach, and uterus [2]. Native Americans with liver, stomach, and kidney cancers have worse survival compared to White people [3], and Hispanics are disproportionately affected by cancers of the uterine cervix [4]. Numerous factors contribute to an individual’s prognosis after a cancer diagnosis, including germline genetics, tumor biology, environment, behavior, health care access and delivery, and social determinants of health [1]. Transparent acknowledgement and discourse of current disparities is a first step in initiating steps to eliminate them across a number of domains. In this review, we will identify health inequities as they relate the evaluation and medical treatment of patients with advanced/metastatic cancer, examine differences in the application of palliative and supportive care interventions, and suggest solutions to promote health equity.

## Health Disparities in Precision Medicine Testing

In the age of precision medicine, increasing emphasis is placed on understanding tumor biology to make educated and evidence-based treatment recommendations. Germline genetic testing, which identifies hereditary cancer conditions [5], and tumor-based somatic testing (next generation sequencing, NGS), which identifies cell signaling pathways for which targeted therapeutics may be available, are both vital to ensuring personalized approaches to care. In fact, genomic sequencing is recommended by the American Society of Clinical Oncology (ASCO) for all patients with metastatic or advanced cancer if the results will guide care [6]. Molecular genotyping has been associated with significantly longer overall survival and better quality of life in patients with advanced stage disease [7]. Despite the importance of these technologies in patient care, however, their utilization is neither universal not equitable across tumor type, race/ethnicity, age, geography, and socioeconomic status.

### Germline Genetic Testing

Substantial variations in germline genetic testing across groups have been reported. In a large review utilizing data from the statewide Surveillance, Epidemiology, and End Results (SEER) registries in California and Georgia, only 6.8% of patients underwent germline testing, varying significantly by disease site, and with substantially fewer Asian, Black, and Hispanic patients undergoing guideline-concordant testing recommendations [8]. When individual cancer types are evaluated, the disparity is even more striking. In a cohort of prostate cancer patients across multiple institutions, racial minorities, those who were non-English proficient, and older patients (≥ 65 years) were less likely to have germline testing [9]. Among patients with ovarian cancer, those with government insurance (Medicare/Medicaid) are > 50% less likely go undergo germline testing [10].

### Somatic Tumor Testing

A number of factors are associated with decreased use of NGS in patients with advanced cancer. Markt et al. reviewed more than 25,000 patients with metastatic colorectal cancer and found that NGS testing occurred less in patients older than 70 years, who were Black or Hispanic, and who had fewer medical co-morbidities [11]. Several authors have reported that among women with breast cancer, OncotypeDx genomic testing occurs less frequently in women who have lower income, are Black and Hispanic, and who live in urban settings [12, 13]. In a study by Press, et al., for those women who did not have OncotypeDx testing, there was a significant increase in chemotherapy receipt across several racial/ethnic groups relative to White women [12]. However, among the patients who had the OncotypeDx testing, there were no differences in chemotherapy receipt by race, suggesting that universal implementation of genomic testing is a means to eliminate treatment disparities. Such treatment implications are not limited to breast cancer, though. Bade et al. [14] evaluated NGS testing completion in patients with acute myeloid leukemia through the lens of area deprivation index (ADI) and insurance status. While the population in this study was too homogenous to draw conclusions about race and ethnicity, the authors report that ADI did not influence NGS testing, but that insurance status did. In fact, patients who were uninsured or insured with Medicare had low testing rates, which resulted in treatment with less aggressive treatment regimens and shorter overall survival. Interestingly, inequity exists even with the specific type of testing NGS used (limited versus extended panel). In a single institutional review of patients across multiple solid tumors, patients with higher socioeconomic deprivation were ~ 30% less likely to have an extended panel and less likely to be matched to a clinical trial [15]. Patients from rural areas were also 15% less likely to have more extensive testing.

In March 2018, Medicare issued a national coverage determination (NCD) for NGS to facilitate increased access. Sheinson et al. [16] performed an evaluation of NGS use pre/post the NCD to determine if this policy was impactful. They found that the NCD was not associated with changes in NGS use by race or ethnicity, either among Medicare beneficiaries or those with other insurance types. Increase in NGS use was appreciated overall, although at a slower rate in African American (14%) and Hispanic patients (23%) relative to White patients.

## Health Disparities in Cancer Treatment

### Chemotherapy

Chemotherapy remains the backbone for treatment in advanced and metastatic malignancies. Disparities in its administration represent a complex interplay of sociodemographics (age, gender, race, and ethnicity), social determinants of health, and geography, and manifest differently according to disease site.

Race and ethnicity are perhaps some of the strongest negative predictors of chemotherapy use in patients with disseminated disease. Among patients with acute lymphoblastic leukemia and acute myeloid leukemia, Hispanics were 20% less likely to undergo hematopoietic cell transplantation (HCT) and non-Hispanic Black patients were 60% less likely to have HCT relative to White patients [17]. Black patients are less likely to get chemotherapy for pancreatic cancer [18, 19], and are given guideline-concordant chemotherapy treatments for lung cancer 22% less often than White patients [20].

Older patients with advanced cancer are also given chemotherapy less often. Wright et al. noted in patients with pancreatic cancer that older patients got chemotherapy at lower rates [19], a finding echoed by Blom et al. among patients with lung cancer [20]. It has been suggested that assessments of frailty be considered in determining the suitability of older patients to undergo chemotherapy, especially in the setting of multiple medical co-morbidities, keeping in mind that age is not always predictive of poor tolerance or outcomes [21]. In fact, Jansen et al. reported on a cohort of stage II and III colorectal cancer patients who underwent chemotherapy and noted that quality of life scores and symptom burden were better in patients ≥ 70 years compared to those aged < 60 years [22].

Gender may also be a determinant in decisions about chemotherapy. Sanford et al. reported that women received chemotherapy 25% less than men after undergoing resection for local pancreatic cancer [18]. Women with bladder cancer are less likely to get chemotherapy, and even when they do, their survival outcomes are worse [23]. When age, gender, and race are considered together, the effect compounds. In a National Cancer Database study evaluating independent predictors for non-recommendation of chemotherapy in women with ovarian cancer, both age ≥ 70 years and Black race were independently association with the recommendation against standard of care chemotherapy. Among Black patients in particular, the recommendation against chemotherapy occurred at a younger age than White patients (64.5 vs. 72 years) and was associated with a lower 5-year overall survival (25.9% vs. 40.3%, *p* < 0.0001) [24].

Marital status also has an association with chemotherapy administration. Wright et al. noted that married patients with pancreatic cancer were more likely to get chemotherapy than those who were not married [19], and Jabo et al. noted that never married patients had a lower likelihood of undergoing HCT for ALL or AML [17]. Klapheke et al. reported that unmarried patients were 40% less likely to get chemotherapy for metastatic bladder cancer [25]. It is unclear if associations with marital status are reflective of social support, financial capacity, or patient decision not to pursue treatment versus actual withholding of treatment. Socioeconomic status, however, is an important determinant of chemotherapy receipt. Klapheke et al. also reported that patients in the lowest socioeconomic status quintile were half as likely to get chemotherapy for metastatic bladder cancer [25]. Insurance coverage predicts chemotherapy use, as well, with uninsured or low income patients with Medicaid receiving treatment at lower rates [18].

Geography and its relationship to care access is also associated with chemotherapy use. Hao et al. reported on a cohort of colorectal cancer patients, noting that the odds of receiving chemotherapy were higher in urban (38%) and suburban (53%) patients than those in rural areas [26]. As a demonstration of intersectionality in this cohort, even though urban living was protective, Black patients in urban settings still received chemotherapy 24% less than White urban patients. Living in rural areas is associated with a high degree of financial hardship, especially when medical therapy requires travel or specialty medication access [27], and likely contributes to the treatment disparity.

### Immunotherapy and Other Novel Therapeutics

There are limited data available about disparities for novel, targeted therapeutics, and in some cases are conflicting. Moore et al. reported on a population of patients with lung cancer treated in a Veterans Health Administration setting, and noted that there were no associations between race and receipt of immunotherapy [28]. In fact, Black patients tended to do better, with fewer immune-mediated adverse events [28]. In contrast, Black patients with human epidermal growth factor receptor 2 (HER-2)-positive breast cancer were 25% less likely to receive trastuzumab, even when accounting for poverty status and comorbidity [29]. Similarly, among metastatic ovarian cancer patients, non-Hispanic Black patients; those who reside in neighborhoods with lower educational attainment; those without health insurance; and those ≥ 65 years of age were less likely to be treated with targeted therapy [30]. In patients with melanoma, immunotherapy is less likely utilized in older patients, patients insured with Medicaid, and patients treated in community settings [31].

Understanding variations in access will be crucial as these medications become increasingly mainstream and the Food and Drug Administration continues to expedite approval for new cancer drugs. A number of potential barriers to universal availability of these life-saving drugs have been identified. Lack of training or experience with novel therapeutics across the entire medical team (physician, nurse, advanced practice provider), limited support for management of toxicities, and geographic access to care are critical staffing and logistical issues [32]. The substantial price of new drugs, variation in cost-sharing by insurance program, and caps on coverage are significant patient concerns, especially since cancer care is associated with significant financial toxicity and increased risk of bankruptcy [32–34].

## Supportive Care and End of Life Treatment

Management of cancer-related symptoms and supportive care is a vital component of cancer treatment. Chemotherapy-induced nausea and vomiting (CINV) is one of the most frequently reported side effects of cancer treatment [35]. Among breast cancer patients, studies have shown decreased use in CINV prophylaxis among Black patients when compared to their White counterparts [36, 37]. Gomez et al. assessed adherence to National Comprehensive Cancer Network guidelines for nausea prophylaxis among lung cancer patients receiving chemotherapy. They found that Black patients and patients of lower socioeconomic status (SES) were less likely than their high-SES counterparts to receive CINV prophylaxis as per guideline recommendations [38]. A retrospective analysis of the SEER database of patients with cancer metastatic to the brain, a condition that is associated with significant symptoms including nausea, found that African- American and Asian patients were less likely to be prescribed anti-emetics than non-Hispanic White patients [39].

Pain is a common symptom reported by cancer patients. Up to 40% of patients with cancer report inadequate pain control [40]. While pain from cancer is not immediately life-threatening, it can cause significant distress and disability, and negatively affect quality of life [41]. Within the American healthcare system, multiple studies have demonstrated that patients from racial minority backgrounds are at higher risk than their White counterparts to receive inadequate treatment for pain [42, 43]. Vallerand et al. evaluated 281 cancer patients treated at a large urban cancer center. They found that Black patients had more pain, higher rates of pain-related distress, and lower functional status as a result of the pain than White patients [44]. In a study of 147 patients undergoing breast cancer chemotherapy, Abujaradeh et al. found that despite controlling for sociodemographic factors including area deprivation index, Black patients had higher pain scores and greater rates of worsening pain over the course of chemotherapy than White patients. The authors concluded that understanding the symptom burden within the context of race prior to treatment could provide a better understanding of the influence of racial disparities on the breast cancer chemotherapy experience [45].

Data on the effect of race and ethnicity on pain management strategies/approaches is mixed. A study of 10,745 pancreatic cancer patients found that race/ethnicity did not independently affect opioid prescribing patterns when sociodemographic factors and tumor characteristics were controlled [46]. In contrast, other studies of oncology patients have found that White patients are more likely to receive pain prescriptions for reported pain. Canick et al. reported that among 25,572 head and neck cancer patients, non-Hispanic White patients were twice than their minority counterparts to receive a prescription for reported pain [47]. Vitzthum et al. looked at 33,872 older adults with cancer and found that when compared to non-Hispanic White patients, non-Hispanic Black patients had significantly lower odds of receiving an opioid prescription for pain; however Asian and Pacific islanders had higher odds [48]. A national Medicare claims data study evaluated disparities within systems of opioid prescription receipt by race. The authors found that within individual health systems, Black and White patients who were served by the same system had very different patterns of opioid receipt with Black patients being less likely to receive prescription treatment. They summarized that these patterns likely reflect both overtreatment of White patients and undertreatment of Black patients [49].

Cancer centers are increasingly providing access to integrative oncology which includes complementary medicine services such as acupuncture and massage as part of multidisciplinary pain management. An Israeli study of ovarian cancer patients found that adherence to an integrative oncology treatment regimen was associated with higher survival rates [50]. It is unclear if the therapy provides survival benefits or if the therapy improves patients’ coping and resiliency in dealing with their cancer. Certain patients may benefit more from these services than others. Booher et al. found that Hispanic patients reported greater expectation of benefit from integrative therapies for pain management [51]. Integrative oncology referrals, however, are not equitably made available. A study of nearly 5000 patients in a large cancer center found that patients’ ethnicity, gender, and age affected referral to integrative oncology services [52].

Palliative medicine is a specialty of medicine that focuses on alleviating symptoms and stress of serious illness. Early palliative medicine integration into oncology care improves patients’ coping with cancer-related stressors, enhances awareness and communication about end of life preferences, and decreases health care utilization at the end of life [53]. Due to its clear benefits, the American Society of Clinical Oncology, the European Society of Medical Oncology, and the National Comprehensive Cancer Network recommend early integration of palliative care for all patients with advanced cancer [54]. In addition to direct clinical benefit for patients, early palliative care consultation in the inpatient setting has been shown to decrease cost of hospital stay [55]. Most terminal cancer patients prefer to die at home [56, 57]. Despite this, many die in hospital settings. Lack of health care resources, low-income status, immigrant status, and non-White race are correlated with higher rates of hospital deaths.

Disparities in cancer treatment persist through end of life. In a study of 1,447 family members of African-American and White cancer patients, family members of African-American descendants were less likely to rate their end of life care as excellent or very good and were more likely to report problematic physician communication or concerns with being informed [58]. Another study of gynecologic cancer patients referred to palliative care found that Black patients were more likely to be seen in the emergency department and more likely to get chemotherapy in the last 30 days of life than White patients [59]. Kohli et al. looked at receipt of palliative care amongst 775,289 patients with metastatic cancer who were White or of Asian American and Pacific Islander (AAPI) descent. When the AAPI group was evaluated as a whole compared to White patients, there were no significant differences in receipt of palliative care. However, when data was disaggregated, differences emerged. Breast cancer patients of Asian Indian/Pakistani descent were less likely to receive palliative care as well as patients of Chinese, Vietnamese, Thai and Asian Indian/Pakistani descent when compared to White patients. Prostate, breast, and lung cancer patients of Japanese and Hawaiian descent were more likely to receive palliative care. This large database study highlighted the differences between racial subgroups and the need for disaggregated research in order to address the unique cultural needs of different groups. Tergas et al. performed multi-institutional study of 302 cancer patients across the United States evaluated the effect of immigrant status on end of life. The authors found that immigrants were more likely to die in hospital settings, less likely to die at their preferred location, and more likely to report poor quality of death [60]. It has been suggested that early palliative care consultation may neutralize some racial/ethnic differences in access to hospice and palliative care services [61].

## Guidelines to Address Treatment Disparities

The disparities presented here are well recognized by a number of oncology societies. Guidelines and recommendations to reduce cancer treatment disparities and enhance health equity have been published by a number of organizations as a way to call attention to the problem and start to address solutions. Representative guidelines from major clinical, population-based, and research societies across the United States, Europe, and globally, are shown in Table 1 [62–67]. Several themes are consistent across the guidelines, speaking to the ubiquity of disparities worldwide. The most notable include:

**Table 1:** Society recommendations to reduce Cancer Treatment disparities and enhance Equity*

Organization	Recommendations
Union for International Cancer Control (UICC)^67^	• Foster patient-centered care that acknowledges and addresses the unique needs and experience across patient populations • Increase funding for cancer research to understand cancer burden, the main disparities in cancer outcomes, and the barriers that prevent certain populations from accessing care even when it is available • Establish robust cancer registries • Design and implement effective cancer control strategies • Enhance health literacy and education around cancer • Address the commercial determinants of health • Implement programs for routine screening of common cancers, and ensure access is available and affordable • Address systemic social determinants of health that impede an individual’s ability to access cancer care
European Society of Medical Oncology (ESMO)^65^	• Promote cancer prevention using adequate knowledge translation tools • Increase diversity in the clinical workforce • Ensure community-level support, including financial plans/aid when patients are on treatment • Enhance technology to facilitate data gathering and sharing, support clinical and shared decision making, and monitor rehabilitation
American Association for Cancer Research (AACR)^63^	• Provide robust, sustained, and predictable funding increases for the US federal agencies and program that are tasked with reducing cancer disparities • Support data collection initiatives • Increase access and participation in clinical trials • Prioritize cancer control initiatives and increase screening for early detection and prevention • Implement policies to ensure equitable patient care • Build a more diverse and inclusive workforce • Enact comprehensive legislation to eliminate disparities (Medicaid expansion, rural health programs, investments in IT infrastructure for underserved communities)
American Cancer Society (ACS)^64^	• Expand health care coverage • Improve access to cancer treatment and palliative care, including prescription drug coverage • Improve data infrastructure • Promote community-engaged research • Increase diversity in the cancer care and research workforce • Permanent guidance on the conduct of decentralized trials
American Society of Clinical Oncology (ASCO)^66^	• Support and promote policies, systems, environments, and practices that address persistent barriers to equitable receipt of high-quality cancer care across the continuum • Protect and promote health care system and payment reforms that improve equity • Promote and encourage culturally and linguistically appropriate, respectful, and high-quality cancer care within all health care systems and organizations • Partner with local communities and legislatures to support the implementation of activities and application of research findings known to improve health equity • Support and equip providers to address disparate health outcomes resulting from institutional discrimination • Develop and dissemination appropriate literacy materials for providers, patients, caregivers, and advocacy group
American Academy of Hospice and Palliative Care^62^	• Focus social justice and health equity work on hospice and palliative care including access to gender affirming care, access challenged/hard to reach populations • Disseminate research, resources, and training to support practitioners in advancing equitable care • Promote and sustain integration of high-quality palliative and hospice care in current and evolving payment and delivery models across all communities and care settings

* Recommendations are summarized and do not represent comprehensive plans

- Improve and expand access to healthcare.
- Increase diversity and representation in the medical workforce.
- Improve data infrastructure and data collection.
- Increase engagement with communities, and particularly those at risk for adverse outcomes of disparate care.

Despite the acknowledgement of disparities in cancer treatment and the development of guidelines to eliminate them, the implementation of strategies to combat disparities has been slow. Multiple levels of influence, including individual, interpersonal, community and societal are at play [68], and in some cases, at odds with one another, thus making comprehensive changes to current patterns of treatment difficult. Resistance to policy improvements is present, given the high cost of implementation strategies that are truly intersectional, even though an estimated $320 billion in excess healthcare spending per year is driven by health disparities [69]. Further, given the diversity of the US population as it relates to socioeconomics, insurers, geography, race, and ethnicity, obtaining granular data to develop personalized or community-level strategies is a monumental task. Fiscella et al. [70] suggested that five main challenges exist to facilitating improvements in healthcare equity, including: (1) failure of buy-in from all participating stakeholders, including payors; (2) absence of granular, high-quality demographic data to guide programs; (3) patient-level concerns about disclosure of demographic information; (4) misuse of patient data; and (5) health care organizational inertia and resistance to change, especially as it relates to understanding race, ethnicity, and socioeconomic disparities. These truly reflect the complexity of the problem, from society to self. A further challenge, though, is within the oncology community itself. The majority of recent efforts related to equitable treatments for advanced and metastatic disease have focused on improved minority participation in clinical trials rather than ensuring access to standard of care treatments. While these are important to ensure that there are good quality data to justify the safe use of novel therapeutics across populations, they fail to address the needs of newly diagnosed patients who are more likely to receive curative intent treatments. Ultimately, collaboration between community partners, patients, healthcare providers, and policy makers will be required to identify opportunities for disparity elimination. Education and continuous quality improvement assessments will also be necessary to ensure that implemented changes are effective, culturally competent, and evolving with the growing oncology landscape.

It is important to appreciate that even though the challenges and proposed solutions are made at the level of medical societies, they are understood fully at the individual and community levels of influence. Snow et al. reported on a qualitative assessment of cancer patients across a number of different disease sites about barriers to care in Canada. Geographic variations in access (both national and regional), lack of funding, physician/patient awareness of options, and social determinants of health were all identified by the patients as significant barriers [71]. Though this is a small study, these findings underscore that the widespread disparities in cancer care are not just present, but are truly the lived experiences of the persons impacted directly by them.

## Conclusion

A number of disparities exist in patients with advanced and metastatic cancer, contributing to inequities in treatment, genomic and genetic analyses, and supportive care practices. These disparities are not limited to one geography or population, and instead are recognized as ubiquitous globally. Race, age, gender, socioeconomic status, immigration status, and area of residence are all identified as factors driving these disparities. As the incidence of cancer continues to increase, and across minoritized populations with fewer resources [72], it is incumbent on the medical community to act now and serve as champions for change. Given the urgent need for progress, in 2024 the World Health Organization published an operational framework for addressing social determinants of health and health inequities [73]. Leveraging guidelines such as these, and those of national cancer societies, is crucial to establish multi-level engagement with stakeholders, from patients to policy makers, and modify the current trajectory of inequities in cancer treatments. Collectively, understanding the complex nature of the problem and committing to its eradication will be key in determining sustainable solutions.

## Key References

1. Alcaraz KI, Wiedt TL, Daniels EC, Yabroff KR, Guerra CE, Wender RC. Understanding and addressing social determinants to advance cancer health equity in the United States: A blueprint for practice, research, and policy. CA Cancer J Clin. 2020;70 [1]:31–46.
  This article is a part of the American Cancer Society’s Cancer Control Blueprint series and provides a framework for understanding and addressing social determeninants of health to promote health equity. It provides recommendations for practice, research, and policy towards this goal.
2. Chakravarty D, Johnson A, Sklar J, Lindeman N, Moore K, Ganesan S, et al. Somatic genomic testing in patients with metastatic or advanced cancer: ASCO Provisional Clinical Opinion. J Clin Oncol. 2022;40:1231-58.
  This Clinical Opinion by the American Society of Clinical Oncology makes clear recommendations that patients with metastatic or advanced vancer should undergo tumor genomic sequencing.
3. Kurian A, Abrahamse P, Furgal A, Ward K, Hamilton A, Hodan R, et al. Germline genetic testing after cancer diagnosis. JAMA. 2023;330:43–51.
  This large study of over 1 million patients across two states found that only 6.8% of patients with cancer underwent germline genetic testing and that minoritized patients were less likely to complete testing than their white counterparts.
4. Ashrafzadeh S, Asgari M, Geller A. The need for critical examination of disparities in immunotherapy and targeted therapy use among patients with cancer. JAMA Oncol. 2021;7:1115-16.
  Immunotherapy and targeted therapies are becoming mainstays of cancer treatment. This viewpoint paper higlights the need to examine disparities in access to these treatments in order to make sure these therapies are distributed equitably among all patients who need them.
5. Boley S, Sidebottom A, Stenzel A, Watson D. Racial Disparities in Opioid Administration Practices Among Undifferentiated Abdominal Pain Patients in the Emergency Department. J Racial Ethn Health Disparities. 2024;11 [1]:416 − 24.
  This study shows that black and hispanic patients were less likely to be treated with opioids when being evalauted in the emergency department for abdominal pain. This contributes to existing data that provider perception of patient pain is affected by patient race.
6. Abujaradeh H, O’Brien J, Mazanec SR, Bender CM, Schlemmer IM, Brufsky AM, et al. The Effect of Race and Area Deprivation on Symptom Profiles over the Course of Early-Stage Breast Cancer. Res Sq. 2023.
  This study of breast cancer patiens undergoing chemotherapy found that while symptom burden increased overtime for all patients, pain and physical function deterioriated at a faster rate for black patients compared to their white counterparts.
7. Morden NE, Chyn D, Wood A, Meara E. Racial Inequality in Prescription Opioid Receipt - Role of Individual Health Systems. N Engl J Med. 2021;385 [4]:342 − 51.
  Using Medicare claims data, this national study found that black patients received signficantly less opioid treatment than white patients for pain management highlighting a racial inequality in pain management.
8. Tergas AI, Prigerson HG, Shen MJ, Dinicu AI, Neugut AI, Wright JD, et al. Association between immigrant status and advanced cancer patients’ location and quality of death. Cancer. 2022;128 [18]:3352-9.
  This study shows that immigrants are more likely to die in hospital settings and less likely to die at their preferred location. Furthermore, immigrants are more likely to have a poor quality of death.
9. AACR Cancer Disparities Progrss Report 2024 Philadelphia: American Association for Cancer Research [Available from: http://www.CancerDisparitiesProgressReport.org/.
  This publication from the American Association for Cancer Research raises awareness of the cancer burden on minority groups and underserved populatons in the United States. It also provides approaches to combat these disparities.
10. World Cancer Day 2024 Equity Report: Union for International Cancer Control; [Available from: https://www.uicc.org/resources/world-cancer-day-2024-equity-report.
  This report published by the Union for International Cancer Control provides tools to promote change, affect policy, and improve healthcare infrastructure to promote equitable cancer care.

## Data Availability

No datasets were generated or analysed during the current study.
